# Supplementary material for: Fate of Pomeranchuk effect in ultrahigh magnetic fields
Source: Nat Commun. 2026 Jan 12;17:367. doi: 10.1038/s41467-025-67053-4 (PMC12796439; doi:10.1038/s41467-025-67053-4)
Supplement: Supplementary file 1 — Supplementary Information [file 41467_2025_67053_MOESM1_ESM.pdf]

Supplementary Materials for

Fate of Pomeranchuk effect in ultrahigh magnetic fields

N. Matsuyama, S. Yokomori, T. Nomura, Y. Ishii, H. Hayashi, H. Ishikawa, K. Matsui, H. Mori, K. Kindo, Y. H. Matsuda, and S. Imajo

1. **Details of the magnetic field profile generated by our pulsed magnets.**
2. **RF impedance measurements.**
3. **Estimation of phenomenological phase diagram based on the Gibbs energy.**
4. **Estimation of changes in sample temperature during pulsed fields.**
5. **Magnetoresistance/impedance data used for the determination of the field-induced transition.**

Supplementary Fig. 1
Supplementary Fig. 2
Supplementary Fig. 3
Supplementary Fig. 4

Reference

1. Details of the magnetic field profile generated by our pulsed magnets.

In this study, we used several pulsed field generation setups. Supplementary Figure 1 exhibits the typical field profile obtained by these pulsed magnets. Among the two non-destructive pulsed magnets used in our measurements, the one up to 60 T with a pulse duration of 36 ms is termed a mid-pulse magnet, while the other can generate 88.6 T with a 3 ms duration is termed a short-pulse magnet (Supplementary Fig. 1a). The destructive magnets utilised at above 100 T region have their pulse duration of μs scale (Supplementary Fig. 1b). For the EMFC system, as the explosion happens at the end of the field-ascending process, the field-descending process cannot be measured.

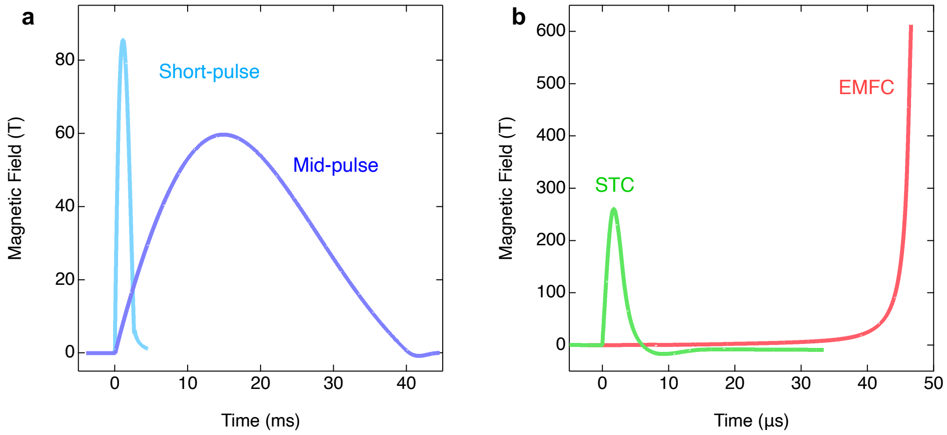

Supplementary Fig. 1 Typical magnetic field profiles generated by (**a**) non-destructive pulsed magnets and (**b**) destructive pulsed magnets.

2. RF impedance measurements.

The RF impedance measurement detects changes in resistance through variations in the reflection wave amplitude and the phase rotation of the injected RF waves [S1]. Supplementary Figure 2a shows a schematic of the measurements. The sample under investigation was placed on a flexible printed circuit (FPC), which was aligned parallel to the magnetic field to minimise induction voltage. A 150 MHz RF sine wave was excited through the first port of a circulator, injected into the sample via the second port, and its reflection was received at the second port before being output through the third port. The reflected signal passed through appropriate filters and was recorded by an oscilloscope. A pickup coil placed beneath the sample detected the magnetic field, and its signal was also recorded via an attenuator. 50 Ω terminators were positioned just before the oscilloscope input to suppress reflections at the terminal and protect the oscilloscope from noise during magnetic field generation.

Ideally, the reflection amplitude exhibits a minimum at the circuit impedance used in the experiments. In this case, it was 50 Ω, while the 0 Ω limit and the insulating limit correspond to the phase rotations of 0 degrees (fixed-end reflection) and π degrees (free-end reflection), respectively. In our experiments, the sample resistance was typically 10 to 50 Ω in the metallic state, whereas the insulating state had a

resistance at least five orders of magnitude higher. Thus, the MITs in $(DMe\text{-}DCNQI)_2Cu$ families were detected as variations in reflection wave amplification and phase rotation through the impedance measurements.

To control the sample temperature, helium flow-type cryostats made of fiber-reinforced plastics were used to prevent induction currents caused by the high sweep rate of the magnetic field. The sample temperature was monitored using a chromel-constantan thermocouple placed near the sample.

Supplementary Figure b show the typical time profiles of the magnetic field and the relative phase change of the RF waves in STC experiments. The severe electromagnetic noise generated by the switching of the high-energy capacitor bank obscured the reflection signal for ~ 1 μs after the start of the field generation. Once the noise subsided, the signal amplitude exhibited a sudden increase at ~ 5 μs, when the magnetic field reached 135 T. At this point, homodyne analysis revealed the phase rotation of the reflected RF waves. These changes together indicate that the sample resistance exceeded the upper detection limit of the impedance measurement, signaling a field-induced MIT. The reflection amplitude and phase remained unchanged for ~ 2 μs, but as the field decreased to ~ 85 T, both values abruptly returned to their original levels observed at zero field.

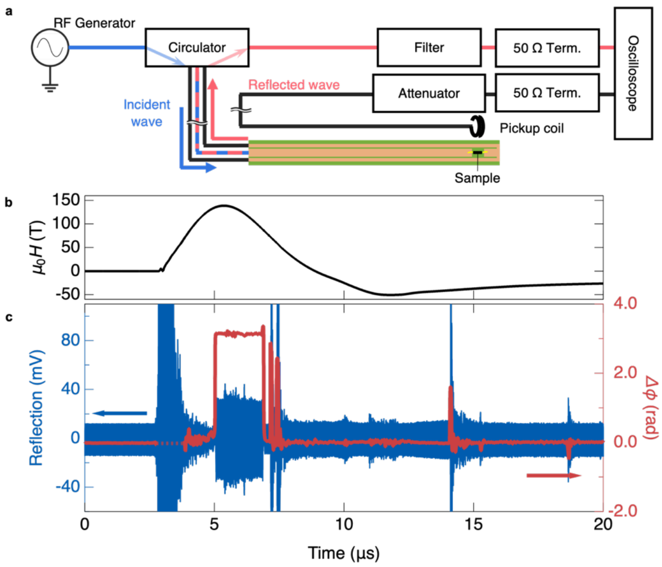

Supplementary Fig. 2 (**a**) Block diagram of the RF impedance measurement setup. (**b, c**) Typical measurement results using STCs. (**b**) Magnetic fields as a function of time. (**c**) Reflection amplitude and phase rotation as functions of time.

3. Estimation of phenomenological phase diagram based on the Gibbs energy.

The pressure-temperature phase diagram of $(DMe\text{-}DCNQI)_2Cu$ [S2] was successfully explained phenomenologically through the discussion of the Gibbs energy. In Fig. 3 (see the main text), we compare the experimentally obtained phase diagram from this study with the phase diagram simulated phenomenologically based on the Gibbs energy.

At the phase boundary, the Gibbs energy of the ordered state equals that of the disordered state. Since Gibbs energy G is defined by the equation, $G = H - TS = \int CdT - T\int (C/T)dT$, it is possible to determine G from the heat capacity C. Regarding the effect of a magnetic field, the work required for an external magnetic field to produce a magnetisation change dM in the system is given by HdM. The area enclosed by the magnetisation curve corresponds to the energy gained by the magnetic field. Therefore, if C and M of the two phases are known, the phase transition point can be determined by identifying where the Gibbs energy difference (ΔG) between the two phases becomes zero.

In this analysis, the contribution of lattice degrees of freedom is neglected, focusing solely on the electronic system. In the Fermi liquid state, the relations $C = \gamma T$ and $M = \chi_P H$, where γ and χ_P represent the Sommerfeld coefficient and the Pauli paramagnetism, respectively, are valid. Note that the bandwidth of (DMe-DCNQI)$_2$Cu is approximately 1 eV [S3]. Since the temperatures and fields in our measurement range are much smaller than this scale, the assumptions $C = \gamma T$ and $M = \chi_P H$ remain reasonable. Here, we assume γ = 20 mJK^{-2}mol^{-1} [S2] and χ_P = 6 mJT^{-2}mol^{-1}, as shown in Supplementary Fig. 3a. Supplementary Figure 3a also demonstrates that S = 1/2 spins on Cu^{2+} in the magnetic solid state exhibit Curie-Weiss behaviour. The magnetisation curve can thus be approximated using the Brillouin function with the Weiss temperature (Fig. 3b). Consequently, the heat capacity in the magnetic solid state can be described by two-level Schottky behaviour. Given that the concentration of Cu^{2+} in the magnetic state of (DMe-DCNQI)$_2$Cu is 1/3 mol due to the formation of the threefold superlattice, the magnetisation saturates at μ_B/3 in the high-field limit (Supplementary Fig. 3b), and the entropy S approaches (Rln2)/3 in the high-temperature limit. Using these simple assumptions, we derived the metal-insulator phase boundary of (d8-DMe-DCNQI)$_2$Cu, which shows good agreement with the experimental results (Fig. 3a).

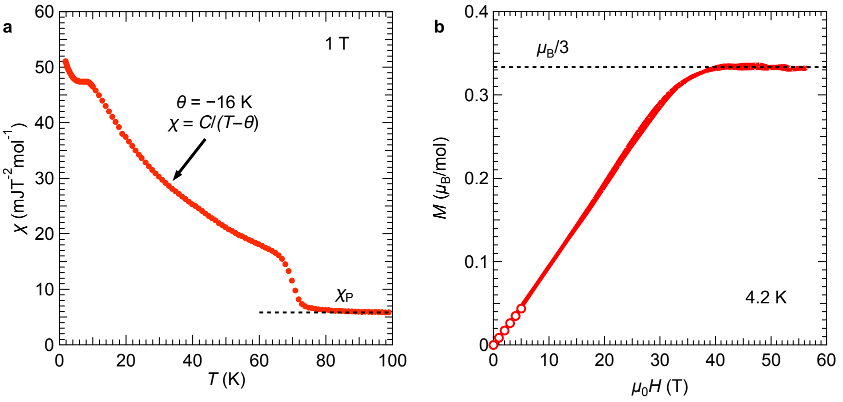

Supplementary Fig. 3 (**a**) Temperature-dependent magnetic susceptibility of (d8-DMe-DCNQI)$_2$Cu at 1 T. Dashed line indicates Pauli paramagnetism in the metallic state. (**b**) Magnetisation curve at 4.2 K. Circles are data points obtained by Magnetic Property Measurement System, while solid curve represent data measured in a pulsed magnetic field. Dashed line represents saturation value of μ_B/3.

4. Estimation of changes in sample temperature during pulsed fields

When conducting experiments in pulsed magnetic fields, if the sample is in a metallic state, eddy currents may generate heat, causing the temperature to rise. The heat generation per unit volume due to eddy currents can be expressed as

$$P = \frac{S}{12\rho}\left(\frac{dH}{dt}\right)^2$$

where ρ is the electrical resistivity, S is the cross-sectional area of the sample perpendicular to the magnetic field, and dH/dt is the rate of change of the magnetic field. When assuming that the pulsed magnetic field with a duration of τ follows a half-cycle sine wave, $H = H_{max}\sin(\pi t/\tau)$ (where $0 < t < \tau$), the temperature increase ΔT is given by the following equation:

$$\Delta T = \frac{\pi^2 S H^2}{24\rho C\tau^2}\left\{t + \frac{\tau}{2\pi}\sin\left(\frac{2\pi}{\tau}t\right)\right\},$$

where C is the heat capacity per volume. In the present experiment, the material is an organic compound with large lattice heat capacity and the measurement temperature is relatively high, resulting in large C. Additionally, since the magnetic field is applied along the one-dimensional direction of the needle-shaped crystal, the cross-sectional area S is significantly small. Therefore, the temperature rise due to the eddy current is expected to be relatively small.

As a rough estimate, assuming $C = 150$ JK^{-1}mol^{-1} [S2], $\rho = 0.1$ mΩcm (Fig. 2a), and $S = 50$ µm*50 µm, in non-destructive experiments (e.g. a 88 T pulsed magnetic field generation with a pulse duration of 3 ms), the temperature rise is only about 2 mK. However, in experiments with destructive magnets, where τ is on the order of microseconds, the eddy-current heating becomes significant. For instance, with a 200 T magnetic field generated in 2 µs, the field sweep causes a temperature rise of 9.3 K above the initial temperature. When the heating effect in destructive-magnet experiments is taken into account, the data obtained connects continuously with the data obtained by non-destructive-magnet experiments, as shown in Fig. 3. Hence, the temperatures shown in the main text are defined as the temperatures at the phase transition points, incorporating the heating effect.

It should be noted that the sample breakage originates from heating induced by metallisation during the insulating–metal transition. If the phase transition proceeded uniformly, it would not result in breakage, as the discussion above indicates that only heating on the order of 10 K would occur. However, the phase transition in this system is first-order and accompanied by a large hysteresis. When a metallic domain begins to form through nucleation, the current density becomes concentrated within that domain, generating a substantial local heating effect. Damage to this region then causes the current density to increase further in the next domain undergoing metallisation, ultimately leading to sample breakage.

5. Magnetoresistance/impedance data used for the determination of the field-induced transition

In Fig. 3a, the transition fields induced by the magnetic field are plotted. Supplementary Fig. 4 shows the magnetoresistance and impedance measurement data for $x = 1$ (a–o), $x = 0.5$ (p–t), and $x = 0.3$ (u–B).

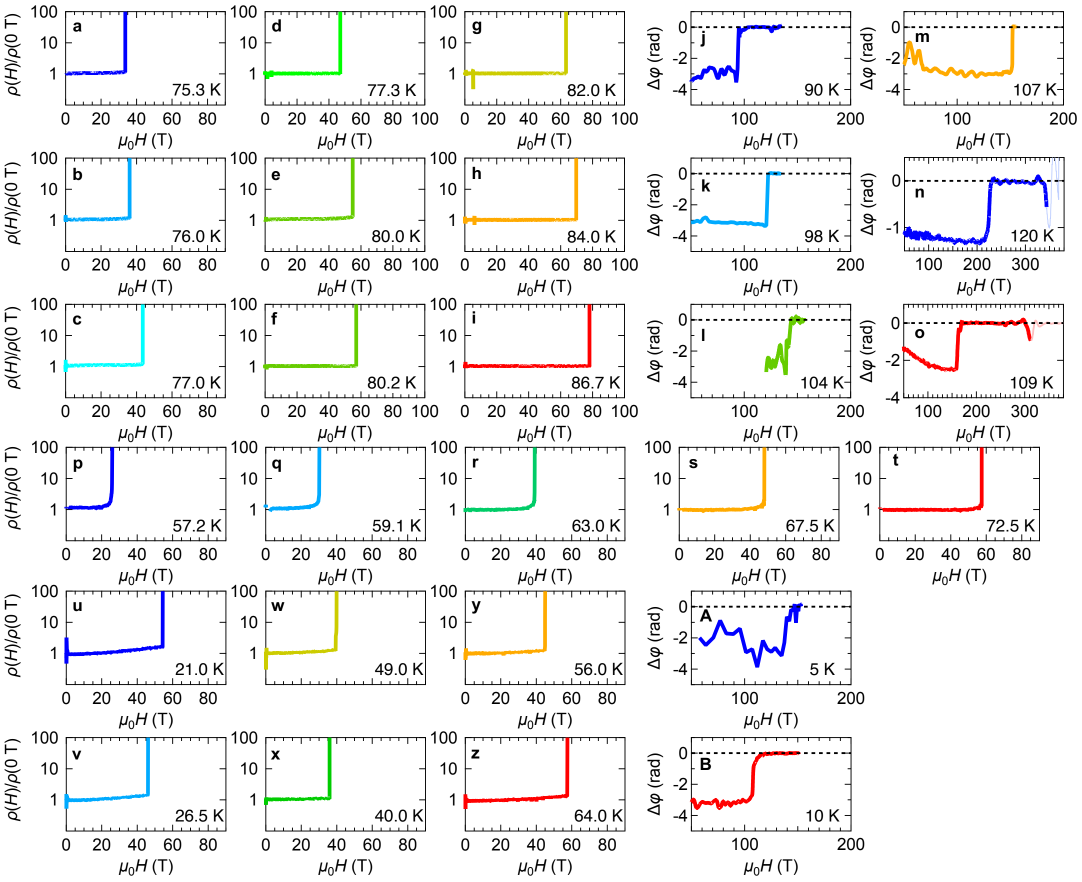

Supplementary Fig. 4 (**a-i**) Magnetoresistance for $x = 1$. (**j-o**) Phase obtained from impedance measurements for $x = 1$. (**p-t**) Magnetoresistance for $x = 0.5$. (**u-z**) Magnetoresistance for $x = 0.3$. (**A**,**B**) Phase obtained from impedance measurements for $x = 1$. Data were collected during field-ascending sweeps.

Reference

S1. Shitaokoshi, T. et al. Radio frequency electrical resistance measurement under destructive pulsed magnetic fields. *Rev. Sci. Instrum.* **94**, 094706 (2023).

S2. Nishio, Y. et al. Thermodynamical Study of (DMe-DCQNI)$_2$Cu System - Mechanism of Reentrant Metal-Insulator Transition -. *J. Phys. Soc. Jpn.* **69**, 1414-1422 (2000).

S3. Uji, S. et al. Coexistence of one- and three-dimensional Fermi surfaces and heavy cyclotron mass in the molecular conductor (DMe-DCNQI)$_2$Cu. *Phys. Rev. B* **50**, 15597 (1994).
